# Does Selection against Transcriptional Interference Shape Retroelement-Free Regions in Mammalian Genomes?

**DOI:** 10.1371/journal.pone.0003760

**Published:** 2008-11-19

**Authors:** Tobias Mourier, Eske Willerslev

**Affiliations:** Ancient DNA and Evolution Group, Department of Biology, University of Copenhagen, Copenhagen, Denmark; Texas A&M University, United States of America

## Abstract

**Background:**

Eukaryotic genomes are scattered with retroelements that proliferate through retrotransposition. Although retroelements make up around 40 percent of the human genome, large regions are found to be completely devoid of retroelements. This has been hypothesised to be a result of genomic regions being intolerant to insertions of retroelements. The inadvertent transcriptional activity of retroelements may affect neighbouring genes, which in turn could be detrimental to an organism. We speculate that such retroelement transcription, or transcriptional interference, is a contributing factor in generating and maintaining retroelement-free regions in the human genome.

**Methodology/Principal Findings:**

Based on the known transcriptional properties of retroelements, we expect long interspersed elements (LINEs) to be able to display a high degree of transcriptional interference. In contrast, we expect short interspersed elements (SINEs) to display very low levels of transcriptional interference. We find that genomic regions devoid of long interspersed elements (LINEs) are enriched for protein-coding genes, but that this is not the case for regions devoid of short interspersed elements (SINEs). This is expected if genes are subject to selection against transcriptional interference. We do not find microRNAs to be associated with genomic regions devoid of either SINEs or LINEs. We further observe an increased relative activity of genes overlapping LINE-free regions during early embryogenesis, where activity of LINEs has been identified previously.

**Conclusions/Significance:**

Our observations are consistent with the notion that selection against transcriptional interference has contributed to the maintenance and/or generation of retroelement-free regions in the human genome.

## Introduction

Transposable elements are genetic elements that are capable of proliferating within–and even between–genomes. The elements can be broadly divided into two classes [1]: Class I elements transpose via an RNA intermediate that is reverse transcribed to DNA. We henceforth refer to class I elements as retroelements. Class II elements transpose via a DNA intermediate. With a few recorded exceptions (e.g. refs [2], [3]) retroelements are found in all eukaryotic genomes examined, and nearly half of the human genome sequence can be attributed to the activity of retroelements [4].

Recently, Simons and colleagues identified almost 1000 regions in the genomes of human and mouse of at least 10 kilo base pairs (kbp) in size with no transposable elements [5]. Such regions–termed TFRs for Transposon-Free Regions–were found to be conserved among other mammals, and associated with microRNAs and genes encoding transcription factors [5], [6]. The authors hypothesized that the TFRs encode regions of essential regulatory information that are intolerant to the insertion of transposable elements. The hypothesised selective disadvantage of transposable elements may in many cases be a result of disruption of the informational content of the sequence in which the transposable element is inserted. Yet, the transcriptional activity of transposable elements could be an additional contributor to the deleterious effects of transposable elements, which are presumably selected against in TFRs. This implies that retroelements are not just avoided in TFRs due to the insertion per se, but also to minimize spurious transcription from retroelements. I.e. it is not necessarily the insertion of a sequence that has a deleterious effect, but rather the subsequent transcriptional activity from the inserted sequence.

Retroelements contain promoters and transcription factor binding sites necessary for their own transcription. Occasionally, the transcription may continue into adjacent regions. If these adjacent regions encode genes, the transcription may potentially result in transcripts containing both transposable element sequence and gene sequence [7], [8], or for example, repress endogenous transcription of the neighbouring gene by promoter competition [9]. Transcriptional interference may potentially occur at different stages of transcription, of which some are experimentally verified and others are purely speculative (see [10] and references therein).

Retroelements display a great divergence in transcriptional capacity and activity. Short interspersed elements (SINEs) contain a weak internal polymerase III promoter [11], usually not capable of initiating transcription by itself [12]. Further, the polymerase III generates only shorter transcripts. In contrast, long interspersed elements (LINEs) and Long terminal repeat (LTR) elements harbour polymerase II transcription start sites that are capable of transcribing into adjacent genomic regions [13], [14]. LINEs even contain an additional promoter situated in the antisense orientation, which is known to transcribe neighbouring genes [15], [16].

The difference in transcriptional features between different transposable elements predicts that the elements will differ in their capabilities in transcriptional interference of neighbouring genes. Firstly, polymerase II transcribed elements will be able to transcribe into adjacent genes, which is not expected for polymerase III transcribed elements. Secondly, as protein-coding genes and presumable microRNAs [17] are transcribed by polymerase II, promoter competition will exclusively be expected from transposable elements transcribed by this polymerase. Thirdly, any physical interaction between transcriptional complexes is expected to be most prominent from polymerase II transcribed elements, simply because these transcriptional complexes will move further along the genome.

Consequently, the impact of transcriptional interference should be highest for LINEs and LTR elements, and we are thus able to test the hypothesis that transcriptional interference is contributing to the existence and maintenance of TFRs: Protein-coding genes and RNA genes that are sensitive to the deleterious effects of transcriptional interference should be enriched in genomic regions devoid of polymerase II transcribed transposable elements, whereas this should not be the case for regions devoid of polymerase III transcribed transposable elements.

Which genes are then susceptible to the deleterious effects of transcriptional interference? Two conditions must be fulfilled. First, the precise regulation of the genes must be crucial to the organism, and second, the space and time (i.e. developmental stage and tissue) of the crucial regulation must coincide with transcriptional activity of the transposable elements.

High levels of retroelement transcriptional activity have been reported in early mouse oocytes embryos [7], [8]. Consistent with this, retrotransposition events have been characterized in the very early stages in mouse development [18], [19]. We would therefore expect genes expressed during, and involved in, early development to reside in genomic regions with limited transcriptional interference by transposable elements. It should be stressed that genetic experiments show that retroelements are capable of retrotransposition in somatic tissue [20]–[22], and hence transcriptional activity from retroelements most likely is not restricted to early development.

Previous work has shown that genes differ in their propensities to harbour retroelements in their genomic vicinity. Sironi and colleagues reported that gene function and expression levels strongly influence the insertion/fixation of retroelements [23]. For example, genes involved in nucleic acid binding and transcription were significantly overrepresented among genes displaying low retroelement densities in their introns [23]. Further, transposable element distributions are found to correlate with recombination rates [24], [25], signifying that multiple factors are shaping the genomic distributions of retroelements. In the present study we exclusively address the temporal expression of genes during development when assessing the impact of transcriptional interference. Clearly, expression profiles during development may be correlated or even functionally related to the genetic features described above.

**Figure 1:**
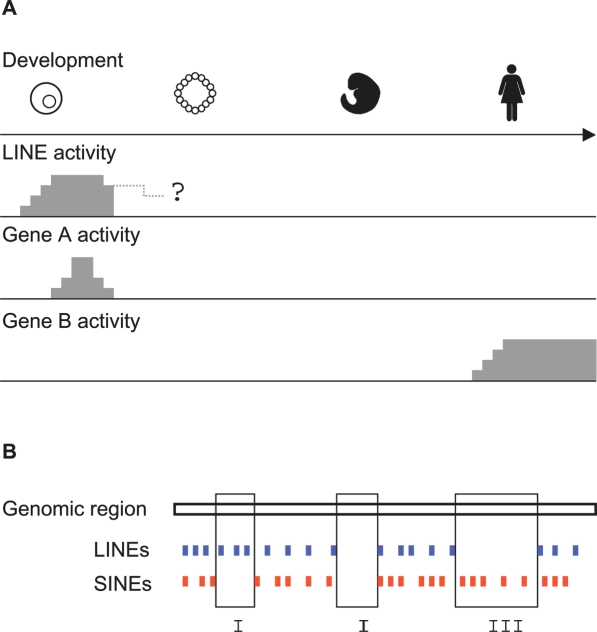
Conceptual background. A) The activity of LINE elements and two hypothetical genes, A and B are shown during human development. LINE activity has been reported during early development, but activity might be present at later stages. Due to transcriptional interference we expect gene A to be under selection for not residing near LINE elements, and consequently genes with expression patterns similar to gene A should be overrepresented in genomic regions devoid of LINEs. In contrast, genes with expression patterns similar to gene D are not expected to be overrepresented in genomic regions devoid of LINEs. Note that the expression of gene A does not necessarily have to be restricted to early development, it is the overlap with LINE activity that is crucial. A further complication is that in later development, activities have to overlap in tissues as well. This issue is not addressed in the figure. B) Outline of retroelement-free region definitions. A hypothetical genomic region is shown with the presence of LINEs (blue) and SINEs (red) shown below. Three regions are boxed. Region ‘I’ does not contain SINEs, but contain LINEs, and is termed SINE-free region in our study. Region ‘III’ does not contain LINEs, but contain SINEs, and is termed LINE-free region. Region ‘II’ contains neither LINEs nor SINEs, and this will not allow us to distinguish features associated with the absence of either LINEs or SINEs. Hence, region ‘II’ is discarded from our analysis.

Our initial analysis prompted us to focus on two types of retroelements, LINEs and SINEs. We recorded regions devoid of LINEs but not SINEs, and vice versa, and analysed the genetic content of these regions. A graphical illustration of the concept is presented in Figure 1. Briefly, since LINEs may cause transcriptional interference, but SINEs will not (or at least at a much lower level), we expect genetic components that are susceptible to transcriptional interference to be overrepresented in LINE-free regions, but not in SINE-free regions.

## Results and Discussion

**Figure 2:**
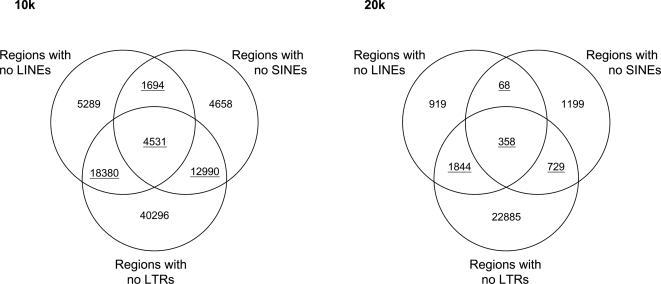
Number of retroelement-free regions in the human genome. Venn diagrams showing the overlap between genomic regions with no LINEs, SINEs and LTR elements, respectively. The intersections show the number of regions overlapping. As this may not be a one-to-one overlap (i.e. one region with no LTRs may overlap two regions with no SINEs), all underlined numbers are average number of overlaps. Numbers for regions of minimum 10 kbp (left) and 20 kbp (right) are shown.

**Figure 3:**
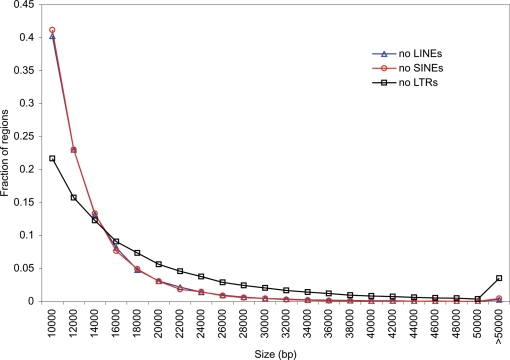
Size distributions of human retroelement-free regions. The size distributions of genomic regions with no LINEs (blue triangles), SINEs (red circles) and LTR elements (black squares) are shown.

We searched the human genome for regions devoid of LINEs, SINEs & LTR elements, respectively. Due to the relative scarcity of LTR elements, the number of regions with no LTR elements is approximately a magnitude higher than regions with no LINEs and SINEs regardless if 10 kbp or 20 kbp is used as a minimum size of regions (Figure 2). Further, the size distribution of LTR-free regions is distinctly different from the LINE- and SINE-free regions, which in turn are remarkably similar (Figure 3). Finally, the evolutionary history of LTR elements is fundamentally different from SINE and LINE elements. While SINEs and LINEs are still actively spreading in the human genome, this is not believed to be the case for LTR element [4]. With a single family as a possible exception [26], the activity of LTR elements may have elements have ceased in the human genome [4]. Considering the above, we decided that the most reasonable comparison would be between regions with no LINEs and regions with no SINEs. To maximize the number of TFRs, we simply ignored LTR elements and focused on LINE-free regions that are not SINE-free (henceforth referred to as LINE-free regions for simplicity), and SINE-free regions that are not LINE-free (henceforth referred to as SINE-free regions) (Figure 1B). We decided to use 10 kbp as size threshold. On average, RepeatMasker annotates app. 5 and 6 LINEs and SINEs per 10 kbp, respectively, and simply by chance we would therefore expect to find a number of 10 kbp regions devoid of LINEs or SINEs. This will inevitably introduce noise to our analysis, although this will only require a higher signal for any biological phenomenon to be detected. Further, given the relatively unknown nature of transcriptional interference and the genomic distance within which it will have an effect, selection against transcriptional interference may work on a relatively small genomic scale. In this case, we are interested in keeping as many LINE-free regions as possible. Using10 kbp as size threshold resulted in 23982 LINE-free regions and 17604 SINE-free regions in the human genome. All subsequent analysis on human retroelement-free regions was performed on these 10 kbp regions. Genomic coordinates are provided in Supplementary Tables S1 and S2.

### Simulated sets of regions

**Figure 4:**
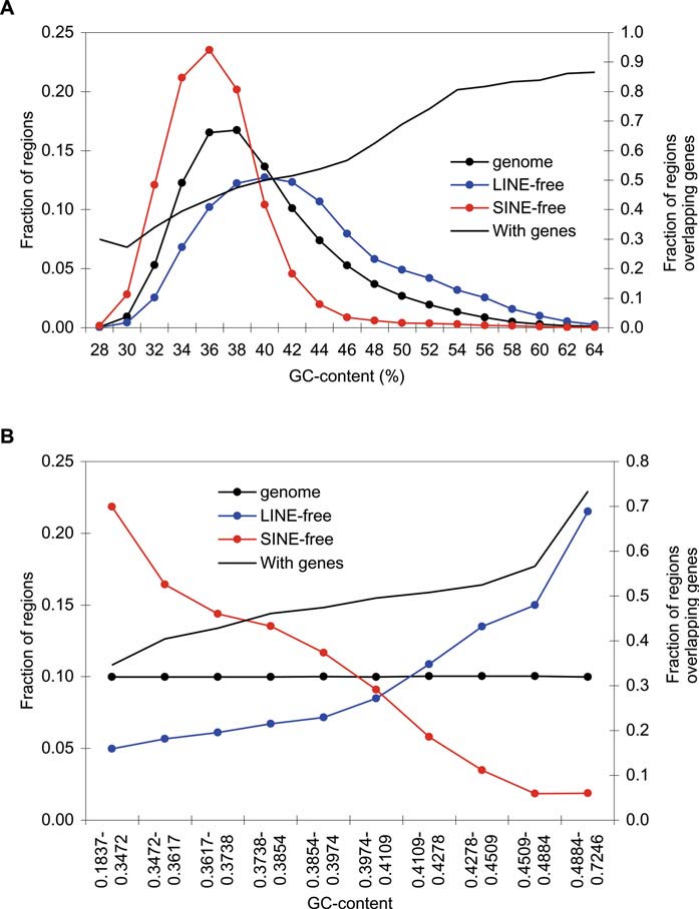
Composition of human LINE- and SINE-free regions. GC-content distributions of LINE- and SINE-free regions, and of the genome divided into 10 kbp sections (left y-axes). Further, the distributions of regions overlapping annotated protein-coding genes are shown on the right y-axes (black lines: “With genes”). A) Composition-value divided genome. B) Composition-size divided genome.

The distributions of LINEs and SINEs in the human genome are characterized by distinctly different compositional patterns, with SINEs predominantly residing in GC-rich genomic regions, and LINEs in GC-poor regions [4], [27], [28]. Not surprisingly, we also find LINE- and SINE-free regions to differ with respect to composition. As gene density and other genomic features are strongly dependent on composition, we need to take this into account when testing if certain features are associated with LINE- and SINE-free regions. We therefore divided the genome into subsets based on GC-content using two different approaches (Figure 4). First, the genome was divided into bins defined by GC-content value thresholds. This way, 19 bins were formed, and we refer to this as the “composition-value divided” genome (Figure 4A). Using GC-content values as thresholds results in a very uneven distribution of the genome in the GC-bins. To make sure that this did not affect the analysis, we also divided the genome into 10 equally sized bins of increasing GC-content. We refer to this as the “composition-size divided” genome (Figure 4B). As expected, LINE- and SINE-free regions display a highly skewed distribution among the subsets (Figure 4). We then used these distributions of retroelement-free regions to construct simulated sets of genomic regions. For example, we constructed 1000 simulated sets of genomic regions, each with approximately the same total size and the same compositional distribution as LINE-free regions. For both LINE- and SINE-free regions we constructed two sets of a 1000 simulations each, one set based on bins from the composition-size divided genome, and another based on the composition-value divided genome. Thus, a total of 4 sets of 1000 simulations were constructed (see Materials and Methods for details). Comparing the LINE- and SINE-free regions against the simulated sets allows the analysis of compositional-independent features of the LINE- and SINE-free regions.

### MicroRNAs

**Figure 5:**
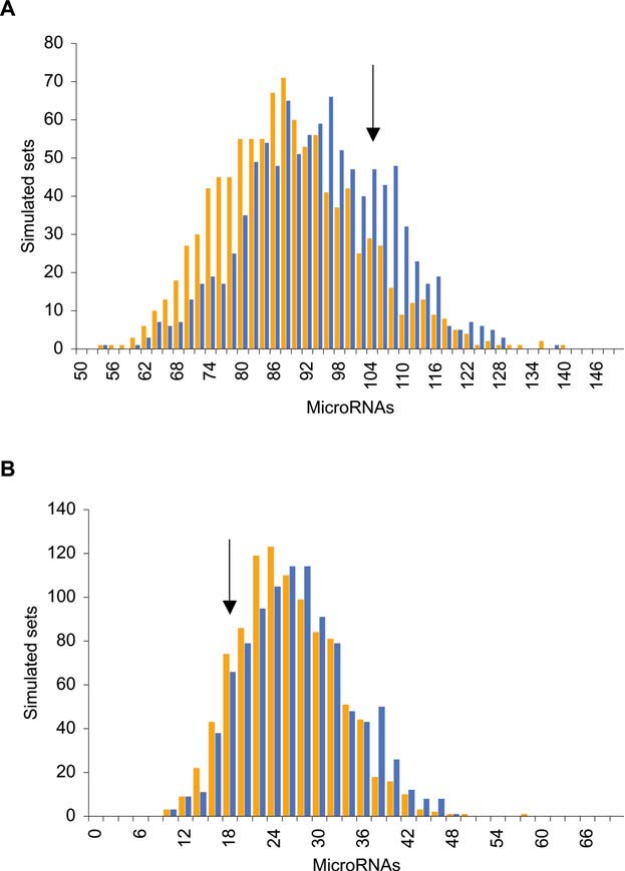
MicroRNAs in LINE- and SINE-free regions. Distributions of microRNA density in simulated sets of human LINE-free regions (A) and SINE-free regions (B). Simulated sets from composition-value divided genome shown as blue bars, composition-size divided genome as orange bars. Observed microRNA numbers in LINE- and SINE-free regions are indicated by arrows.

MicroRNAs are small (21–23 nucleotides) RNAs involved in gene regulation (see [29] for review), and key regulators of plant and vertebrate development [30], [31]. Simons et al. [5] reported an overrepresentation of microRNAs in TFRs. When assuming a random distribution of microRNAs, the presence of 105 microRNAs in LINE-free regions is highly significant using a binomial distribution (expected number of microRNAs = 62; p<10^−6^). SINE-free regions contain only 18 microRNAs, which is in fact lower than expected (expected number of microRNAs = 45). However, when compared to 1000 simulated sets of regions with a similar compositional distribution, both the density of microRNAs in LINE- and the density in SINE-free regions fall well within the range of the densities of the simulated sets (Figure 5). Due to our procedure for constructing simulated sets, these are slightly smaller than the real LINE- and SINE-free regions (see Materials and Methods). However, as the simulated sets are only app 0.14% and 0.56% smaller than the real LINE- and SINE-free regions, respectively, we consider this difference as having no impact on the observed patterns. Similar results are obtained when exclusively analysing non-intronic microRNAs (not shown). Hence, when adjusting for compositional biases we do not observe any significant association between microRNAs and LINE- and SINE-free regions.

### Protein-coding genes overlapping LINE- and SINE-free regions

**Figure 6:**
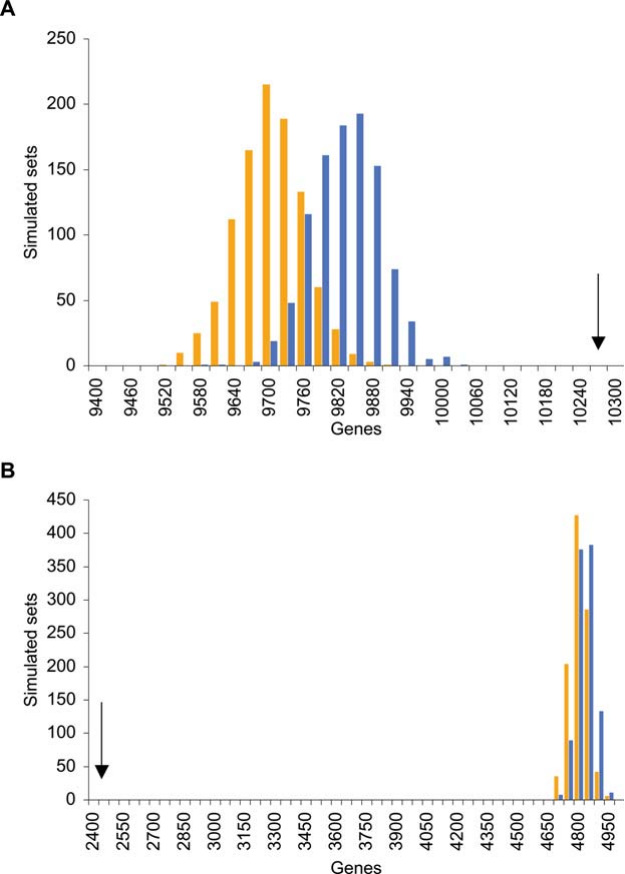
Genes in LINE- and SINE-free regions. Distributions of gene density in simulated sets of human LINE-free regions (A) and SINE-free regions (B). Simulated sets from composition-value divided genome shown as blue bars, composition-size divided genome as orange bars. Observed gene numbers in LINE- and SINE-free regions are indicated by arrows.

LINE- and SINE-free regions overlap 10281 and 2411 protein-coding genes, respectively. Compared to the number of genes expected from a random distribution (2300 and 1689, respectively), the observed numbers of genes are significantly higher (binomial distribution; p<10^−6^ in both cases). When corrected for compositional biases using the simulated sets (see above), we find that the number of genes in LINE-free regions is still significantly higher than expected (Figure 6A). In strong contrast to this, SINE-free regions overlap a significantly lower number of genes than expected from the composition of the regions (Figure 6B).

### Repeat content of LINE- and SINE-free regions

A considerable fraction of the SINE-free regions consist primarily of other types of repetitive DNA. This is not the case for LINE-free regions (Supplementary Figure S1). Such highly repetitive SINE-free regions are unlikely to contain genes, microRNAs and other genetic components, which may affect the analysis. We therefore performed the above analysis on gene and microRNA density on the subset of SINE-free regions with less than 50% repetitive sequence (as denoted by RepeatMasker). This did not change the observations that SINE-free regions contain fewer protein-coding genes than expected, and that the number of microRNAs in SINE-free regions does not deviate from random expectations (Supplementary Figure S2).

### Developmental expression of genes associated with retroelement-free regions

To assess the gene expression patterns during embryogenesis, we turned to mouse, where expression data are abundant from all developmental stages. Using the same procedure for defining LINE and SINE-free regions in the human genome, we found 35433 and 27436 LINE- and SINE-free regions, respectively, in the mouse genome [32]. The numbers of mouse LINE- and SINE-free regions are thus both around 1.5 higher than in humans. Genomic coordinates for the mouse LINE- and SINE-free regions are provided in Supplementary Tables S3 and S4. From the Mouse Genome Informatics Database [33], [34] (www.informatics.jax.org), we downloaded all available gene expression data with annotated developmental stage. This resulted in expression data for 6466 mouse genes representing 27 different developmental stages. We were able to retrieve human homologues for 5546 of the mouse genes.

**Figure 7:**
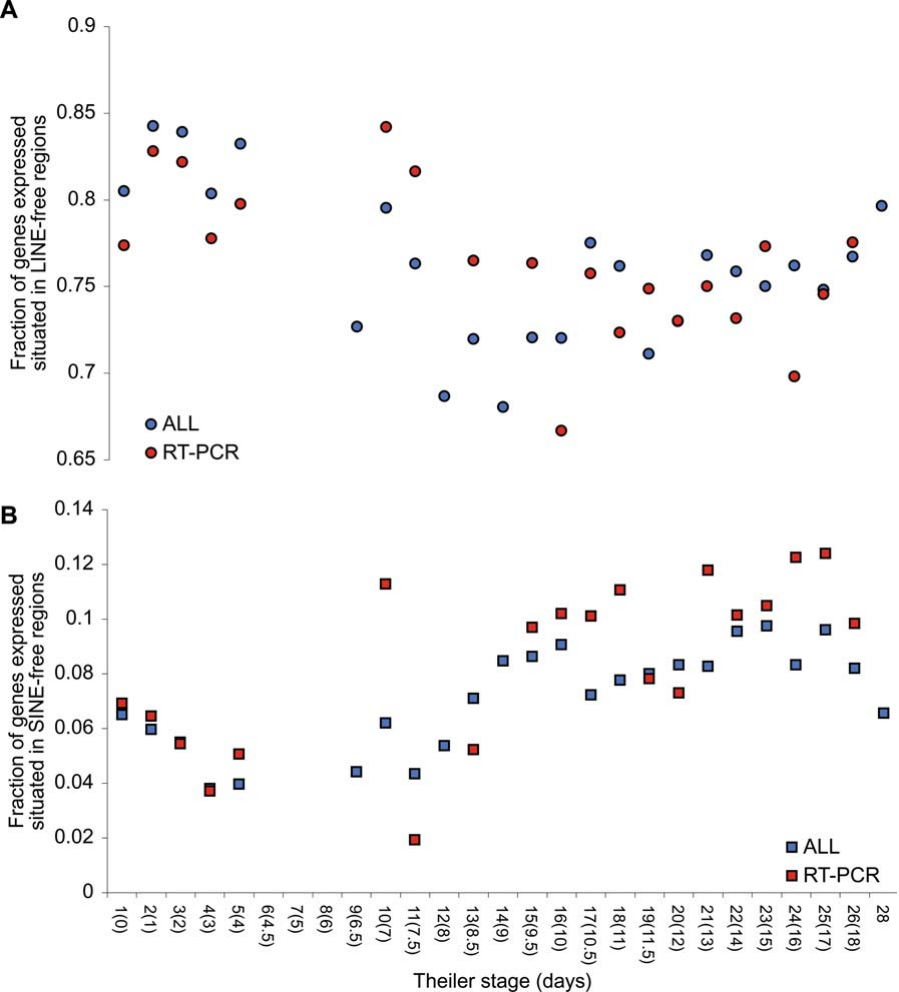
Developmental expression of mouse genes overlapping retroelement-free regions. For 27 developmental stages, the fraction of mouse genes with detectable expression that overlap LINE-free regions (A) and SINE-free regions (B) is plotted. Data for the earliest stages are predominantly stemming from RT-PCR experiments (red circles and squares). For completeness, combined data for all experiments are shown as blue circles and squares. For both RT-PCR and all experiments, only stages with at least 100 detectable genes were recorded. Timing and description of stages are adopted from the Edinburgh mouse atlas project (http://genex.hgu.mrc.ac.uk/). Theiler stage numbers [52] are shown with the corresponding number of days in parenthesis. Selected description of stages are: 1) One-cell egg, 2) Dividing egg, 3) Morula, 4) Blastocyst, 7) Implantation, 13) Turning of the embryo, 28) Post-natal.

**Figure 8:**
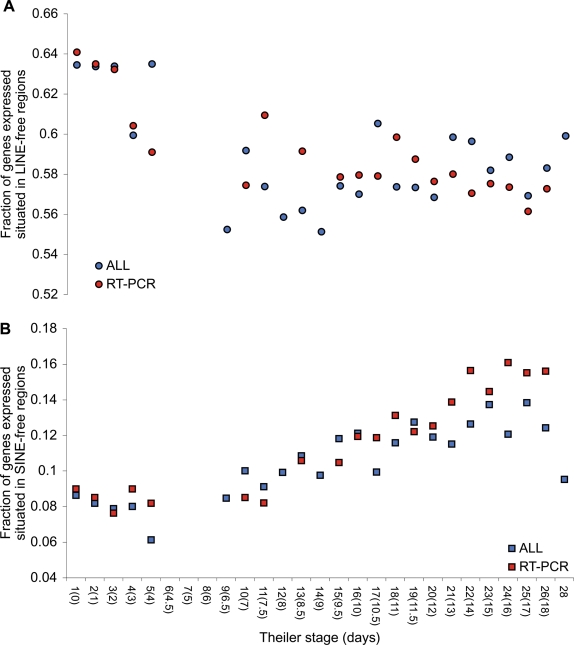
Developmental expression of human genes overlapping retroelement-free regions. For human homologues of mouse genes with detectable expression, the fractions that overlap LINE-free (A) or SINE-free (B) regions are shown as in Figure 7.

**Figure 9:**
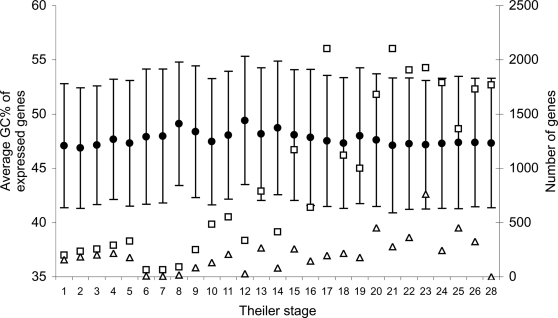
Gene features during development. The average GC-content for mouse genes expressed during embryo development shown as filled circles (left axis). Error bars correspond to plus/minus one standard deviation. Theiler stages as in Figure 7. The number of mouse genes in our data set being expressed for each stage is shown on the right y-axis. Squares: All detection methods; Triangles: RT-PCR detection only.

To estimate the activity of genes residing in LINE- and SINE-free regions during development, we recorded the fraction of genes expressed at a given development stage that are residing in retroelement-free regions (Figure 7). If a mouse gene is expressed at a given stage, we assumed the same activity of the human homologue (Figure 8). The gene expression calls from the Mouse Genome Informatics Database come from a range of molecular techniques. As detection by RT-PCR constitutes the majority of data from very early development (Figure 9) we have plotted data derived from RT-PCR analysis alone as well as data from all techniques in Figure 7 and 8. Previous studies have reported transcriptional activity from retroelements in mouse oocytes and 2-celled embryos [7], [8]. Interestingly, blocking reverse transcriptase activity in murine embryos results in developmental arrest at the two- and four-cell stages [35], [36]. Based on this, we would expect a higher fraction of genes active in pre-blastocyst stages to be residing in LINE-free regions. In fact, we do observe a decline in the fraction of genes in LINE-free regions being expressed from pre-blastocyst to post-natal stages (Figure 7A and 8A) in both mouse and human. In contrast, an opposite trend of increasing expression during development is observed for genes associated with SINE-free regions (Figure 7B and 8B). The fact that genes associated with SINE-free regions increase in expression during development could be a simple consequence of the decrease of genes associated with LINE-free regions. Approximately 80% of mouse genes and 60% of human homologues with detected expression during development are associated with either LINE- or SINE-free regions (not shown), However, we cannot rule out that the causal relationship between the two patterns works in the other direction, and that the increase in the fraction of genes expressed being associated with SINE-free regions reflects a true biological phenomenon.

Given the compositional differences between LINE- and SINE-free regions, the opposing trends in Figure 7 and 8 could be explained by a compositional shift in genes expressed during development. As seen from Figure 9, the average GC-content of expressed mouse genes is unchanged during development, and hence the different expression patterns of genes residing in LINE- and SINE-free regions cannot easily by explained by a simple shift in composition among active genes.

Using a hypergeometric distribution and after Bonferroni correction only human homologues in SINE-free regions as detected by RT-PCR from late development (Theiler stages 10, 11, 13, 15–22, 24–26 & 28) were found to be expressed significantly higher than expected by chance (not shown). This pattern of significance was not observed when using all detection methods, nor was it observed for mouse genes. So in general, expression levels of genes in LINE- or SINE-free regions are rarely significant for individual stage samples. However, when testing expression levels from early stages versus late stages, these were found to be significantly different in all cases (Supplementary Table S5). As Theiler stages 6–8 were omitted due to too few data in all cases analysed, this was chosen as the border between early and late stages. Hence, early stages were defined as Theiler stages 1–5, and late stages as Theiler stage 9 or later.

The apparent decrease in the number of genes associated with LINE-free regions suggest that if this is a result of selection against transcriptional interference from LINEs, either i) LINE activity decreases from early development to the post-natal stages, or ii) that the selection is reduced during development, or iii) that the selection is restricted to a subset of cells, and hence less likely detected as the number of cells and tissues increases.

### HOX genes and retroelements

A group of genes with expected intolerance to transcriptional interference from retroelements are the HOX genes. HOX genes encode transcription factors that regulate vertebrate body plan formation during development [37], and are also involved in adult tissue maintenance [38]. Especially in early development, the inadvertent up- or down-regulation of HOX-genes could be fatal to the organism (e.g. [39], [40]). HOX genes reside in gene clusters [41], and these clusters are in fact associated with remarkably low levels of transposable elements [4], [5].

**Figure 10:**
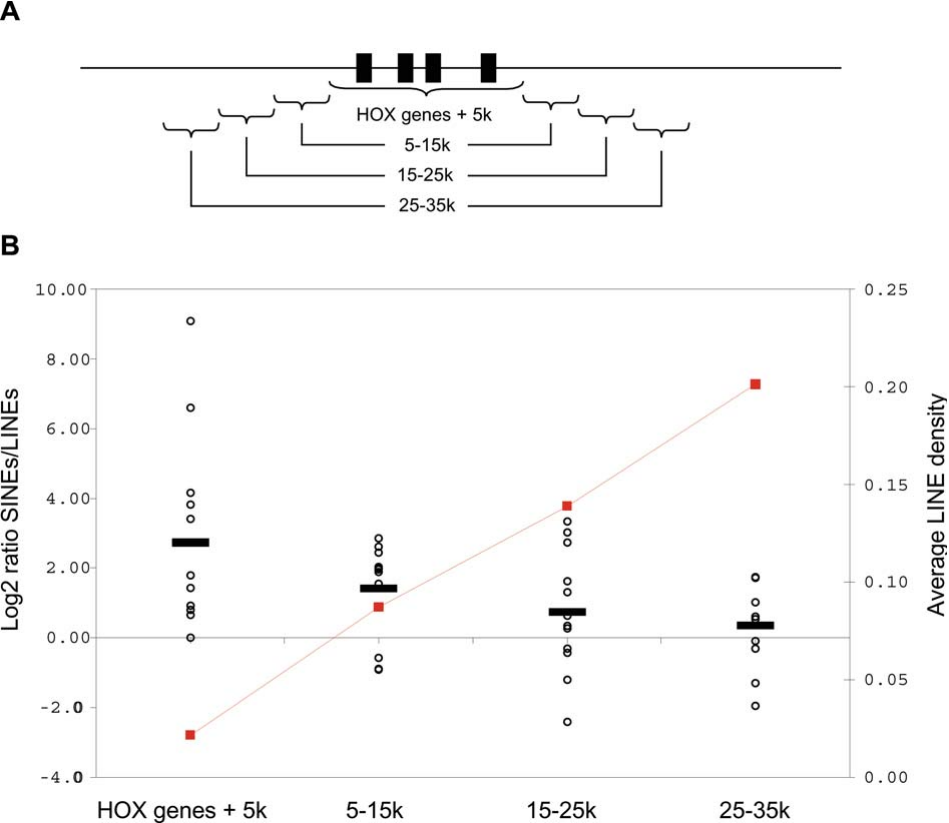
LINE and SINE density around HOX-gene clusters. A. Schematic display of neighbouring regions around HOX-gene clusters. HOX genes residing less than 20 kbp apart were grouped together as a cluster, and the surrounding regions were compared for LINE and SINE sequences. B. Log2 ratio between SINEs and LINEs (bp/bp) plotted on the left y-axis for the 4 genomic regions surrounding HOX gene clusters (as shown on the x-axis). Values for the 12 HOX clusters shown as open black circles and averages are shown as black bars. Using wilcoxon matched-pairs test, all regions except ‘HOX genes+5k’ versus ‘5–15k’ and ‘15–25k’ versus ‘25–35k’ were found to be significantly different at the 0.02 confidence level (not shown). Absolute LINE densities (LINE bp/region bp) for the regions are plotted as red squares on the right y-axis.

We found that our human LINE-free regions did not overlap with more HOX-genes than would be expected by chance (data not shown). This may not come as a surprise as our LINE-free regions are defined as regions without LINEs not overlapping regions without SINEs, and HOX genes are generally found to reside in regions devoid of any transposable element [4], [5]. Nevertheless, if we compare the ratio between SINE and LINE sequence around the HOX-gene clusters, we find that the closer we move towards the HOX genes the fewer LINE sequences relative to SINE sequences we observe (Figure 10). Further, as the average absolute density of LINEs increases with distance from HOX gene clusters, the change in ratios is not explained by changes in SINE densities (Figure 10). Although HOX-genes do not specifically reside in LINE-free regions, this suggest that in terms of transposable elements HOX genes display some preference for elements not capable of transcriptional interference (*i.e.* a preference for SINEs over LINEs).

### Concluding remarks

We have addressed the hypothesis that transcriptional interference from retroelements is a contributing factor in creating and maintaining transposon-free regions in the human genome. To test this, we focused on regions devoid of LINEs (capable of transcriptional interference) and regions devoid of SINEs (not capable of transcriptional interference), and surveyed the genetic content of these regions. It should be stressed that transcriptional interference cannot alone be responsible for the existence of TFRs, as selection against transcriptional interference will only affect the elements that are capable of inducing transcriptional interference. Hence, the distribution of SINEs cannot be explained by selection against transcriptional interference. The hypothesis on selection against transcriptional interference can thus be seen as an additional contributor to the uneven genomic distribution of certain retroelements.

For both LINE- and SINE-free regions we found no enrichment for microRNAs. This may not be in conflict with the idea of retroelement transcriptional interference: in zebra-fish expression of microRNAs is not detected before 16 hours after fertilization [42], corresponding to the segmentation period, roughly 10 hours after the blastula period [43]. Therefore, microRNA expression potentially post-dates the putative high levels of retroelement transcriptional activity in early development, in which case no selection is expected against microRNA location near retroelements.

Correcting for compositional biases, we found that LINE-free regions are enriched for protein-coding genes, whereas the opposite is found for SINE-free regions. This corresponds well to previous observations of element densities around genes [27], and is consistent with a general selection against transcriptional interference, although this does not address whether LINEs are specifically selected against among genes that are expressed during periods of known LINE activity and require precise regulation. This is, however, indicated by the observation that during early developmental stages, a higher fraction of active genes are overlapping LINE-free regions.

Other genetic features that differ between LINEs and SINEs could in principle be responsible for the observed differences between LINE- and SINE-free regions (and the genes they overlap). For example, methylation of retroelements could silence nearby genes, and the methylation levels of retroelements may differ. In human germ cells, Alus (the dominant human SINE) are protected from methylation by the binding of a sperm protein (which does not happen in somatic tissue) [44]. However, a study where germ line methylation levels were assessed from CpG-to-TpG substitutions (as the methylation target CpG is prone to deamination to TpG when methylated), showed that although the pattern of methylation differed between LINEs and SINEs, the overall levels of methylation were comparable [45]. This, together with the fact that it is not known if mouse SINEs are similarly protected against methylation, makes it unlikely that divergent methylation patterns are responsible for the observed differences between LINE- and SINE-free regions.

In summary, we have provided observations supporting the hypothesis of selection against transcriptional interference, although rigorous testing will require more knowledge of retroelement activity. Due to their sequence redundancy retroelements–and transposable elements in general–are most often discarded from large-scale expression studies. Relative few reports of transpositional activity exist, although activity has been detected both during early development and in somatic tissue [18], [21], [22], [46]. Further, there may be a high degree of transcriptional activity from retroelements that does not result in transposition, as exemplified by the antisense promoter of L1 LINEs [15], [16]. Hence, a better description of human retroelement transcriptional activity in space and time is a prerequisite in assessing the interplay between retroelements and the general genomic architecture.

## Materials and Methods

Regions devoid of LINEs, SINEs and LTR elements were extracted using the RepeatMasker [47] annotation of the human (hg18) and mouse (mm9) genome at The UCSC Genome Browser [48], [49]. Sequence gaps were treated as repeats. Simons and co-workers removed all regions that were believed to stem from genomic duplication events or from transfers of sequences from the mitochondrial genome [5]. However, as the aim of our study was to analyse the content of the retroelement-free regions, and not to establish their existence, all regions were analysed regardless of potential differences in their evolutionary history.

Annotations of ENSEMBL protein-coding genes [50] were retrieved from BioMart (www.biomart.org). Data and genomic coordinates for microRNAs were downloaded from miRBase [51]. From the Mouse Genome Informatics Database we retrieved lists of genes with detected expression in Theiler stages 1 through 26, as well as stage 28. A description of the Theiler stages [52] can be found at the Edinburgh Mouse Atlas (http://genex.hgu.mrc.ac.uk/Databases/Anatomy/MAstaging.shtml). Conversion between mouse and human genes was performed using the orthology data from BioMart (www.biomart.org).

### Simulated sets

The genome was divided into non-overlapping sections of 10 kbp in size. The GC-content was then recorded for each region, and according to this, the sections were assigned to GC-intervals (either composition-size or composition-value defined, see main text). Next, the LINE-free regions were mapped to the genomic non-overlapping sections. All sections with at least 5 kbp covered by a LINE-free region were recorded along with their corresponding GC-interval. Simulated sets were constructed by randomly drawing the same number of non-overlapping 10 kbp sections in each GC-interval as the number of sections covered by LINE-free regions. This was done similarly for SINE-free regions. As the size of the simulated sets was thus restricted to an integer of 10 kbp, a slight size difference exists between simulated sets and the real LINE- and SINE-free regions. The simulated LINE sets are 333330 Mb in size, or approximately 0.14% smaller than the real set (333816 Mb). The simulated SINE sets are 243790 Mb in size, or approximately 0.56% smaller than the real set (245155 Mb).

### HOX genes

HOX gene annotations were retrieved from the ENSEMBL gene descriptions. Based on genomic coordinates 72 HOX genes were grouped into tentative gene clusters if the genes were less than 20 kbp apart. This resulted in 12 gene clusters. For the regions surrounding these clusters we recorded the number of base pairs being occupied by either LINEs or SINEs, and calculated the log2 ratio between the two.

## Supporting Information

Figure S1 (0.06 MB PDF)

Figure S2 (0.09 MB PDF)

Table S1 Human LINE-free regions
(0.57 MB TXT)

Table S2 Human SINE-free regions
(0.42 MB TXT)

Table S3 Mouse LINE-free regions
(0.85 MB TXT)

Table S4 Mouse SINE-free regions
(0.65 MB TXT)

Table S5 (0.02 MB DOC)
