# Supplementary material for: Does Selection against Transcriptional Interference Shape Retroelement-Free Regions in Mammalian Genomes?
Source: PLoS One. 2008 Nov 19;3(11):e3760. doi: 10.1371/journal.pone.0003760 (PMC2582637; doi:10.1371/journal.pone.0003760)
Supplement: Figure S1 — (0.06 MB PDF) [file pone.0003760.s001.pdf]

Supplementary Figure S1

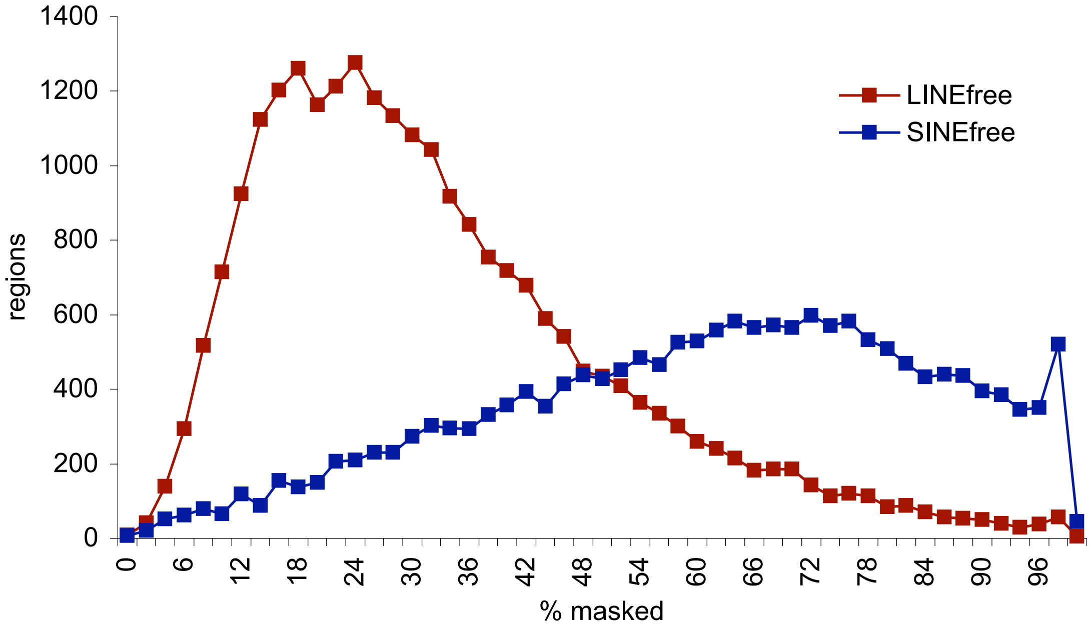


**Figure S1.** Repeat content in human retroelement-free regions
For each retroelement-free region the percentage of sequence being masked by RepeatMasker was recorded. For SINE- and LINE-free regions the distribution of masking percentages are shown.
