# Supplementary material for: Does Selection against Transcriptional Interference Shape Retroelement-Free Regions in Mammalian Genomes?
Source: PLoS One. 2008 Nov 19;3(11):e3760. doi: 10.1371/journal.pone.0003760 (PMC2582637; doi:10.1371/journal.pone.0003760)
Supplement: Figure S2 — (0.09 MB PDF) [file pone.0003760.s002.pdf]

Supplementary Figure 2

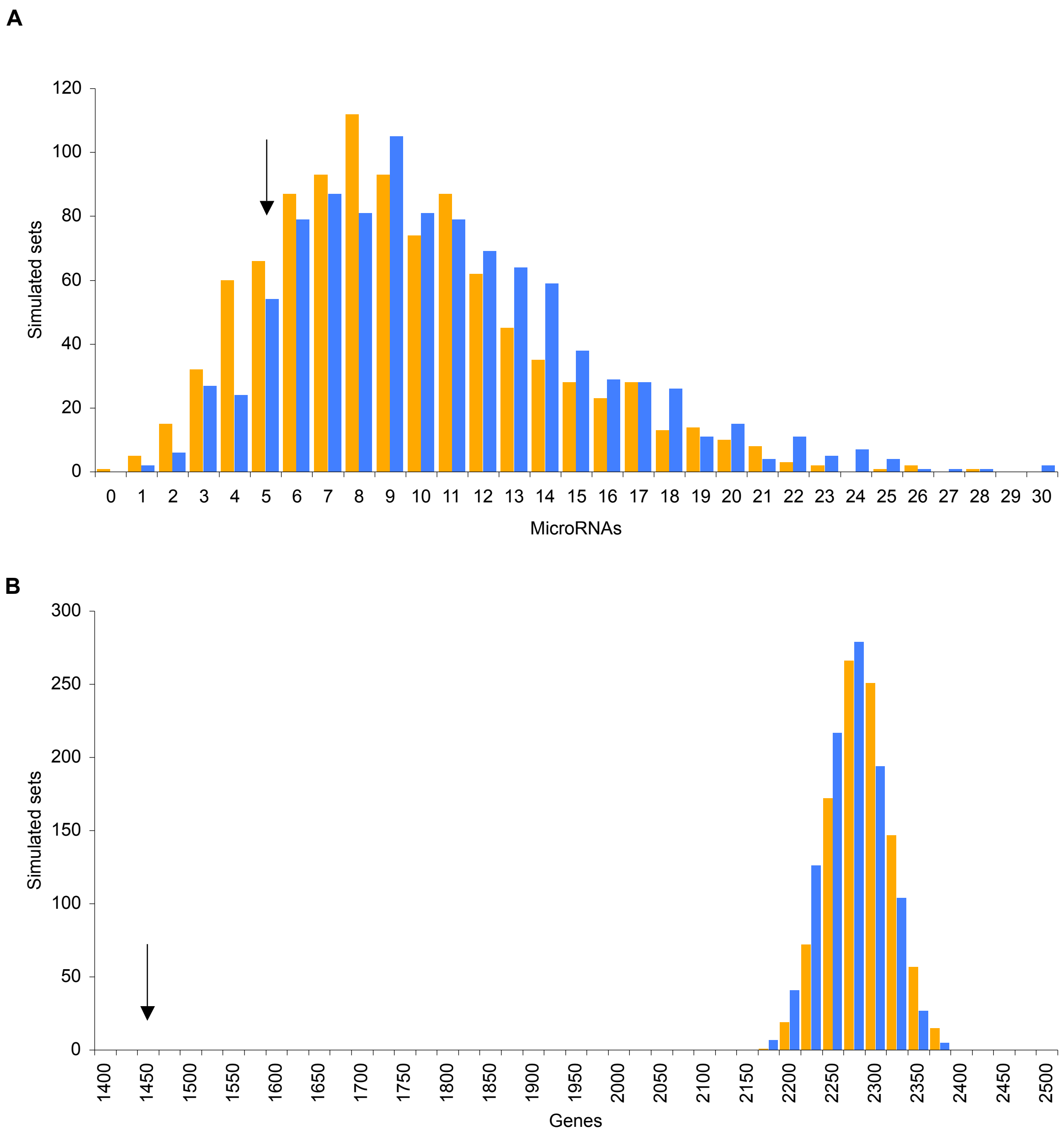

Figure S2. MicroRNAs and genes in repeat-poor SINE-free regions
Distributions of microRNA (A) and gene densities (B) in simulated sets of human SINE-free regions.
Only SINE-free regions with less than 50% masked sequence were considered, both in real and in simulated sets.
Arrows indicate the observed microRNA and gene numbers in the real data.
