# Supplementary material for: Does Selection against Transcriptional Interference Shape Retroelement-Free Regions in Mammalian Genomes?
Source: PLoS One. 2008 Nov 19;3(11):e3760. doi: 10.1371/journal.pone.0003760 (PMC2582637; doi:10.1371/journal.pone.0003760)
Supplement: Table S5 — (0.02 MB DOC) [file pone.0003760.s007.doc]

Supplementary Table S1

|  | method^a^ | P^b^ |
| --- | --- | --- |
| Mouse |  |  |
| genes in LINE-free regions | ALL | 4.7×10^-5^ |
|  | RT-PCR | 1.1×10^-2^ |
| genes in SINE-free regions | ALL | 5.3×10^-3^ |
|  | RT-PCR | 7.7×10^-3^ |
| Human |  |  |
| genes in LINE-free regions | ALL | 9.4×10^-5^ |
|  | RT-PCR | 1.5×10^-3^ |
| genes in SINE-free regions | ALL | 9.4×10^-5^ |
|  | RT-PCR | 2.4×10^-3^ |
|  |  |  |

Notes:

Mann-Whitney U test for difference in fraction of genes expressed overlapping retroelement-free regions between early (Theiler stages 1-5) and late (Theiler stages 9-28).

a) Detection method of expression. Either all methods (ALL) or the subset from RT-PCR only (RT-PCR).

b) Two-tailed probability
